# Patterning of the Vertebrate Head in Time and Space by BMP Signaling

**DOI:** 10.3390/jdb11030031

**Published:** 2023-07-03

**Authors:** Kongju Zhu, Herman P. Spaink, Antony J. Durston

**Affiliations:** 1 Institute of Biology, Leiden University, Sylviusweg 72, 2333BE Leiden, The Netherlands; 2 Department of Pathology, Brigham and Women’s Hospital, 60 Fenwood Road, Boston, MA 02115, USA; 3 Department of Genetics, Harvard Medical School, Boston, MA 02115, USA; 4 Division of Genetics and Genomics, Boston Children’s Hospital, Center for Life Sciences, Blackfan Circle, Boston, MA 02115, USA

**Keywords:** BMP signaling, head patterning, extreme anterior domain (EAD), Xenopus

## Abstract

How head patterning is regulated in vertebrates is yet to be understood. In this study, we show that frog embryos injected with Noggin at different blastula and gastrula stages had their head development sequentially arrested at different positions. When timed BMP inhibition was applied to BMP-overexpressing embryos, the expression of five genes: *xcg-1* (a marker of the cement gland, which is the front-most structure in the frog embryo), *six3* (a forebrain marker), *otx2* (a forebrain and mid-brain marker), *gbx2* (an anterior hindbrain marker), and *hoxd1* (a posterior hindbrain marker) were sequentially fixed. These results suggest that the vertebrate head is patterned from anterior to posterior in a progressive fashion and may involve timed actions of the BMP signaling.

## 1. Introduction

During early development, the vertebrate embryo is patterned from anterior to posterior in a temporally progressive manner [1,2,3,4,5]: anterior tissues are specified early, and more posterior tissues are determined progressively later. Whereas coordination between temporal and spatial control of anterior-posterior (A–P) patterning is evident, a thorough understanding of the underlying mechanisms is still lacking in vertebrates.

In frogs, a BMP/anti-BMP dependent time-space translation mechanism has been proposed for trunk-tail patterning by Hox genes [6,7]. In this mechanism, Hox genes are sequentially activated in a high BMP region of the mesoderm (non-organizer mesoderm) [8], where their expression is dynamic and unstable. As the mesoderm involutes during gastrulation, Hox expressing cells are successively exposed to signals from the Spemann organizer, resulting in the Hox sequence being fixed at different points along the forming axis. In this way, the timing information encoded by Hox genes is translated into a spatial pattern. The putative organizer signals that stabilize Hox codes are BMP antagonists, e.g., Noggin [9] and Chordin [10], because these mimic the function of the organizer in dorsalizing the embryo [11,12], inducing a secondary axis [10,13,14], and rescuing A–P axes in ventralized embryos [7,10]. Notably, timed Noggin treatments in ventralized embryos not only rescue the A–P axis, but also the spatial pattern of Hox gene expression [7]. This conclusion is further supported by a recent study in chicks, which reported the fixation of Hox codes in the explanted posterior primitive streak (containing high BMP mesoderm) by Noggin treatments [15]. Together, these findings suggest that BMP signaling is involved in patterning the trunk-tail part of the axis by (directly or indirectly) regulating Hox gene expression.

In the rescue experiments mentioned above [7,10], Noggin and Chordin treatments can also rescue the head part of the axis, suggesting that BMP signaling may also be involved in patterning the head. Using heat-shock inducible *chordin* transgenic lines (Tg (hsp70:chd)), Hashiguchi et al. have shown in zebrafish that the expression of *six3* (a forebrain marker) [16], *otx2* (a forebrain and mid-brain marker) [17,18], *gbx1* (the counterpart of Xenopus *gbx2*; a rostral hindbrain marker) [19], and *hoxb1b* (a caudal hindbrain marker) [20], are sequentially expanded by timed anti-BMP treatments from mid-blastula to early gastrula stages [21]. This is consistent with the observations that timed Noggin injections in ventralized embryos rescued different portions of the A–P axis in frog [7], and that progressively later anti-BMP treatments resulted in progressively more posterior axis defects in zebrafish [22]. These findings raise an interesting question: is BMP signaling involved in progressively patterning the head (brain)?

In the deuterostome embryo, the front-most portion of the A–P axis is not the head, but the extreme anterior domain (EAD), a region wherein ectoderm and endoderm directly juxtapose [23]. In frogs, this region gives rise to three organs, the cement gland (CG), the primary mouth, and the anterior pituitary [24]. Among them, the cement gland is an ectodermal organ that lies anterior to any neural tissue [25]. The formation of CG can be affected by perturbations of the development of the dorsal mesoderm (the Spemann organizer) [26,27], suggesting a requirement for organizer signals in the formation of this anterior-most structure. It would therefore be interesting to see if CG formation is also regulated by BMP signaling.

To test the role of BMP signaling in head patterning, we performed timed anti-BMP treatments in both wild-type (WT) and ventralized frog embryos. This resulted in sequential arrest (in WT embryos) or rescue (in ventralized embryos) of head patterning at different values, suggesting that a timing mechanism, which is BMP dependent and can be converted into spatial patterns by anti-BMP signals, may be involved in patterning the vertebrate head.

## 2. Materials and Methods

### 2.1. Microinjection

Frog embryos were harvested from naturally mated females and staged according to Nieuwkoop and Faber [28]. For timed anti-BMP treatment in wild-type embryos, 200 nL of 0.1 µg/µL human noggin protein (Sigma-Aldrich, H6416, Zwijndrecht, The Netherlands) was injected into the blastocoel of embryos at stage 8, 9,10, 10.5 and 11, respectively. The embryos were then cultured to stage 28 for taking pictures. A similar approach has been used by others [7,29]. These experiments were repeated at least three times and more than 80 embryos were used for each time point. mRNA for injection was transcribed with mMessage mMachine Kit (Ambion, Life technologies, AM1340, Carlsbad, CA, USA) from the following plasmids after linearization at the appropriate restriction sites: *pSP64T-BMP4* (for BMP4 RNA) [30], and *pCS2-hSmad6GR* (for smad6GR RNA) [31]. To induce full ventralization, about 2 ng of BMP4 RNA was injected to each embryo at 2-cell or 4-cell stage and cultured to stage 26. Timed anti-BMP treatment in BMP-ventralized embryos was achieved by using a combined injection of 2ng BMP4 RNA and 2ng smad6GR RNA at the 2-cell or 4-cell stage. The embryos were then treated with 10 µM dexamethasone for 2 h at the desired stages and cultured to stage 26. For induction of dosalization, 2 ng of smad6GR RNA was injected at the 2-cell or 4-cell stage and the injected embryos were treated with 10 µM dexamethasone for 2 h at stage 7. These experiments were repeated at least three times and >40 embryos were used for each condition.

### 2.2. Whole Mount In Situ Hybridization

When the desired stages were reached, embryos were fixed overnight in MEMFA at 4 °C. After dehydration in 100% methanol, they were stored in methanol at −20 °C until use. Whole mount in situ hybridization (WISH) was performed as previously described [7]. The probes for in situ hybridization were synthesized from the following plasmids after linearization: *pVZ1-xcg1* (for *xcg*-1 probe) [32], *pBSSK-Six3* (for *six3* probe) [33], *pBluescript-KS-xotx2* (for *otx2* probe) [34], *pXgbx-2* (for *gbx-2* probe) [35], and *pBluescript SK-xHoxLab1* (for *hoxd1* probe) [36].

## 3. Results

### 3.1. Timed Anti-BMP Treatment Arrests Head Patterning at Different Positions

**Figure 1:**
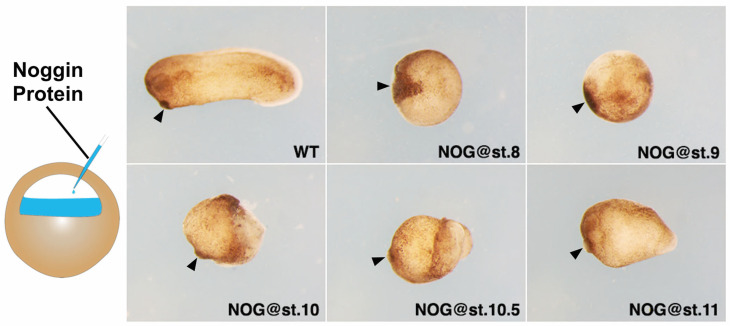
Timed Noggin-injection in wild-type embryos resulted in progressive arrest of head patterning: 200 nL of 1 ng/µL Noggin was injected into the blastocoel of the embryo at different stages. Anterior is to the left and dorsal is up. Black arrows point to the position of the cement gland. These experiments were repeated at least three times and more than 80 embryos were used for each time point.

To examine the role of BMP signaling in head formation, we injected Noggin protein, an antagonist of BMP [9,37], to the blastocoel of the embryo at stage 8, 9, 10, 10.5 and 11 (from blastula to gastrula stage) and cultured them to stage 26 (Figure 1). From stage 8 to 10, the injected Noggin protein mainly affects the ectoderm and the future neurectoderm. At stage 10.5 and 11, the anterior tip of the involuting mesoderm will also be affected. Embryos injected with Noggin at stage 8 formed a ball of tissue with a large cement gland. Embryos injected at stage 9 also showed a blob of tissue, but the cement gland was much smaller. When Noggin was injected at st.10, a visible, short head (half head) was formed in the embryo. Morphologically, injection of Noggin at st.10.5 and 11 resulted in progressive formation of more posterior structures.

### 3.2. Timed Anti-BMP Treatment in Ventralized Embryos Rescued Different Portions of the Head

**Figure 2:**
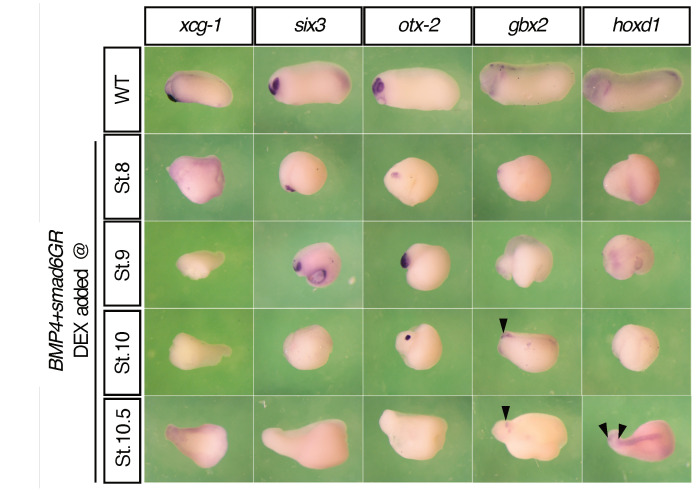
Timed anti-BMP treatments in ventralized embryos led to sequential fixation of anterior genes. The expression of *xcg-1*, *six3*, *otx2*, *gbx2*, and *hoxd1* in *bmp4*-injected embryos that were subjected to Smad6 treatment at different stages. These experiments were repeated at least three times and >40 embryos were used for each condition. Black arrows indicate the anterior borders of the expression domains.

The above observations are supported by gene expression analysis. In zebrafish, timed Chordin treatments from mid-blastula to mid-gastrula stages sequentially expanded the expression domains of *six3*, *otx2*, *gbx1*, and *hoxb1b* [21], suggesting that timing is involved in head patterning and is likely to do with BMP signaling. We therefore postulate that the “head timer” is BMP-dependent and can be sequentially fixed by BMP inhibition, resulting in positional values being sequentially specified. To test this hypothesis, we conducted timed anti-BMP treatments in ventralized frog embryos (high BMP) (Figure 2).

**Figure 3:**
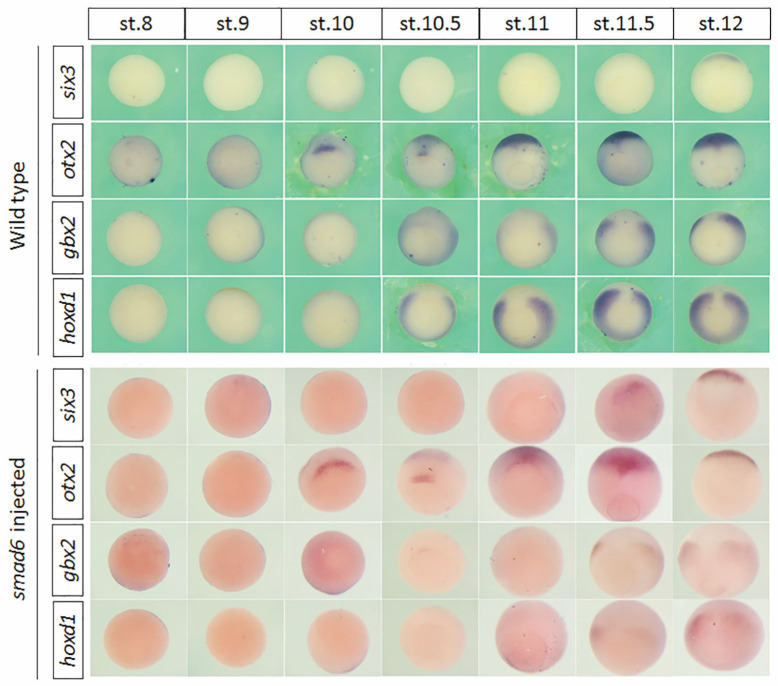
The expression of *six3*, *otx2*, *gbx2* and *hoxd1* at different stages in wild-type and *smad6*-injected embryos. Embryos are vegetal views with dorsal to the top. These experiments were repeated at least three times and >40 embryos were used for each condition.

In Xenopus, ventralization can be achieved by BMP overexpression [38,39,40,41], while dorsalization can be achieved by BMP inhibition [11,42]. In our experiments, injection of the frog embryo with 2ng *bmp4* resulted in complete ventralization, showing a blob of tissue that had no axis (Figure S1). When the embryo was injected with the same amount of *smad6*, an inhibitory Smad that can interfere with BMP pathway [43,44,45], however, it displayed a dorsalized phenotype (Figure S1). We then did anti-BMP treatments in BMP4-ventralized embryos at different stages using a Smad6GR construct [31], which is inducible by dexamethasone. Timed Smad6 inductions fixed five anterior markers sequentially: it strongly fixed *xcg-1* at stage 8, *six3* at stage 8 and 9, *otx2* at stage 9 and 10, *gbx2* at stage 10 and 10.5, and *hoxd1* at stage 10.5 (Figure 3).

### 3.3. The Timing of A–P Markers Is Disrupted in smad6-Injected Embryos

During trunk patterning, collinearity causes Hox genes to be expressed in a 3′ to 5′ order, and that more 3′ genes are expressed earlier and more anteriorly than/to more 5′ ones [46,47,48]. The temporally collinear expression of Hox genes has been proposed to serve as a timer, which can be interpreted and translated into spatial patterns [7]. Since the anterior genes are sequentially fixed earlier than Hox genes by anti-BMP treatment (Figure 2), it is thus interesting to see if these genes are also expressed in a temporal sequence which can complement the Hox sequence to constitute an integrative timer. Therefore, we next examined the endogenous expression of these anterior genes at different stages in wild-type embryos (Figure 3). Although these genes showed a spatial sequence of expression along the A–P axis (Figure S2), their activation did not strictly follow this spatial arrangement. For example, *six-3* demarcates the most anterior border of the developing neural plate [49], but it was expressed at the end of gastrulation, much later than the other genes. The expression domain of *gbx2* is directly anterior to that of *hoxd1* but they were expressed at similar times. Moreover, unlike Hox genes, which are expressed in ventral and lateral mesoderm during gastrulation, the earliest expression of *six3* and *otx2* was located on the dorsal side of the embryo. The expression kinetics of these genes make some of them less likely to be “timer genes” themselves. Even so, however, the timing of their expression was disrupted by *smad6* injection (Figure 3). For example, the expression of *six3* in *smad6*-injected embryos was advanced to stage 11.5 from stage 12. Expression of *gbx2* and *hoxd1* was significantly reduced and only detectable from stage 11.5, whereas their expression in WT embryos was observed earlier (at stage 10.5). Although the time of *otx2* expression was not affected, its expression domain was slightly expanded at stage 10.

## 4. Discussion

During animal development, the patterning of the trunk-tail part of the axis has been known to be regulated by Hox genes [48,50,51]. The regulation is likely to involve timed interactions between BMP and anti-BMP during gastrulation [6,7]. In this study, we show that BMP and anti-BMP may also be involved in regulating head patterning in the developing frog embryo.

When the inhibitor of the BMP pathway, Noggin protein, was applied to the frog embryo from the mid-blastula to mid-gastrula stage, the patterning of the head was arrested at different positional values, i.e., cement gland, forebrain, mid-brain, hindbrain, and neck (Figure 1). Sequentially, later Noggin treatments arrested head formation at more and more posterior positions, suggesting that the EAD and head are patterned gradually in a timed fashion. Similar effects of BMP intervention have also been observed during trunk-tail patterning. In zebrafish, sequential BMP inhibition at later stages results in axial defects at progressively posterior positions, i.e., in the trunk and tail [22]. Together, these results imply that the process involved in head-tail patterning can be stopped sequentially by timed BMP inhibition.

The timing observed In Figure 1 fits well with a recent finding in zebrafish, Ich showed sequential expansion of head markers (*six3*, *otx2*, *gbx2*, and *hoxb1b*) by timed Chordin treatments [21]. We extended this study by performing timed anti-BMP treatments in *bmp4*-overexpressing embryos (Figure 2). This sequentially rescued the expression of *xcg-1*, *six3*, *otx2*, *gbx2* and *hoxd1*, suggesting that the patterning of the EAD and head involves timed BMP/anti-BMP interactions. Alternatively, the gradual rescue of these anterior marker genes could possibly be explained by the distance of different head structures to the source of BMP or anti-BMP signals. However, since there is no evidence so far showing a BMP gradient running from anterior to posterior in the head, it is more likely that a temporal component is involved.

It is worth noting that, Ioth in Hashiguchi’s study and in this study, the last and most posterior component of the head gene sequence is *hox1*:*hoxb1* and *hoxd1*, respectively. Since *hox1* is the most anterior component of a previously elucidated Hox time-space sequence, the spatial arrangement of these early induced head genes is clearly complementary to and continuous with the later, more posterior Hox gene sequence. There is also evidence that timed Noggin treatments in ventralized embryos at different gastrula stages sequentially fixes Hox gene expression [7], suggesting that similar mechanisms may be involved in patterning the expression of Hox genes and genes expressed more anteriorly. However, unlike Hox genes, only a subset of these anterior head genes was expressed sequentially, i.e., *otx2*, *gbx2* and *hoxd1* (Figure 3). Though, a finer time scale may be needed to separate the expression of *gbx2* and *hoxd1*. Other gene like Six3 are less likely to regulate timing by itself. It is more likely to be regulated by an upstream BMP-dependent timer gene. Nevertheless, the expression of all the examined genes was disrupted in BMP-inhibited embryos (Figure 3), suggesting that they can respond to BMP interventions. Moreover, the time at which some of these genes are expressed is also of crucial importance to embryo development. For example, Gbx2 shows a significant effect on head development when ectopically expressed at stage 9 and 10. The effect gets less drastic when it is expressed at later stages, e.g., stage 12 and 13 [52]. Together, these results further emphasize the importance of BMP signaling in regulating head patterning.

**Figure 4:**
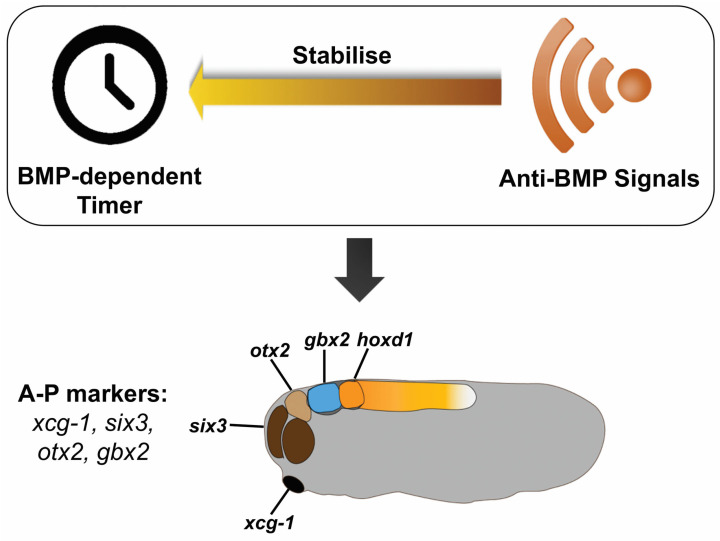
Hypothesis for head patterning by BMP signaling. A currently unknown, BMP-dependent timer is running during head patterning. The timer can be fixed by anti-BMP signals and regulates the expression of anterior genes: *xcg-1*, *six3*, *otx2*, *gbx2*, and *hoxd1*, resulting in the head being progressively patterned.

Although the mechanism by which head patterning is regulated by BMP signaling still needs to be elucidated, the results in this study suggest two aspects of head patterning. First, the vertebrate head is patterned in a temporally progressive manner: the EAD is patterned first, followed by patterning of the forebrain, midbrain, hindbrain, and the neck. Second, BMP signaling is involved in patterning the head in time and space, which can be seen from the progressive arrest of head formation by timed anti-BMP treatments (Figure 1) and from sequential fixation of anterior marker genes (*xcg-1*, *six3*, *otx2*, *gbx2*, and *hoxd1*) by anti-BMP (Smad6 induction) in BMP-injected embryos (Figure 2). Based on these results, here we hypothesize that a similar mechanism to that patterns the trunk-tail part of the axis may also operate during head patterning [6,7] (Figure 4). A key component of this mechanism is a BMP dependent timer, which is a temporal gene sequence. The expression of the (putative) “timer genes” occurs in a high BMP environment in a transient, come-and-go manner. Once the cells are exposed to anti-BMP signals during morphological cell movement, the “timer genes” will be fixed, meaning that the cells will keep the “timer genes” expressed at that moment and prevent the expression of later “timer genes”. Therefore, in BMP-injected embryos, timed BMP inhibition (by Smad6) only stabilizes a subset of positional markers that correspond to specific head regions. For example, induction of Smad6 at stage 8 mainly stabilized *xcg-1* (EAD), and *six3* (forebrain), and slightly *otx2* (midbrain); induction at stage 9 stabilized *six3* (forebrain) and *otx2* (midbrain), but not *xcg-1*; and induction at stage 10 stabilized *otx2* (midbrain) and *gbx2* (hindbrain), but not *xcg-1* and *six3* (Figure 2). This suggests that “the timer” still runs in BMP-embryos, but due to lack of stabilization by anti-BMP signals, the “timer genes” are only expressed in a transient manner. Timed anti-BMP treatments stabilize/fix genes expressed at the corresponding time points and stop the timer from running further, leading to the formation of specific regions, manifested by the expression of specific marker genes. In WT, however, both the timer (BMP dependent) and the stabilizer (anti-BMP signals) are present in the embryo. The ticking of the timer and the stabilization occur concomitantly. Therefore, BMP inhibition will fix the timer and lead to the formation of structures up to the fixation point rather than structures only at the fixation point. The essence of the timer that regulates head patterning is yet to be elucidated but may involve a subset of genes examined in this study, i.e., *otx2*, *gbx2* and *hoxd1*. Further studies are needed to test our hypothesis further and to uncover how the head patterning process is regulated by BMP signaling. This study, as the basis and first step of our hypothesis, argues that the vertebrate head is progressively patterned, and the patterning process is likely to be regulated by BMP signaling.

## Data Availability

The data presented in this study are available in this article and its associated supplementary materials.

## Supplementary Materials

The following supporting information can be downloaded at https://www.mdpi.com/article/10.3390/jdb11030031/s1: Figure S1: Ventralization and dorsalization induced by *BMP4* and *smad6* injection, respectively; Figure S2: The expression of *six3*, *otx2*, *gbx2* and *hoxd1* at stage 15. Embryos are dorsal views with dorsal to the top.

## References

[1] Eyal-Giladi H. (1954). Dynamic Aspects of Neural Induction in Amphibia. Arch. Biol..

[2] Gamse J., Sive H. (2000). Vertebrate Anteroposterior Patterning: The Xenopus Neurectoderm as a Paradigm. Bioessays.

[3] Gamse J.T., Sive H. (2001). Early Anteroposterior Division of the Presumptive Neurectoderm in Xenopus. Mech. Dev..

[4] Nieuwkoop P.D. (1952). Activation and Organization of the Central Nervous System in Amphibians. Part III. Synthesis of a New Working Hypothesis. J. Exp. Zool..

[5] Stern C.D., Charite J., Deschamps J., Duboule D., Durston A.J., Kmita M., Nicolas J.F., Palmeirim I., Smith J.C., Wolpert L. (2006). Head-Tail Patterning of the Vertebrate Embryo: One, Two or Many Unresolved Problems?. Int. J. Dev. Biol..

[6] Durston A.J., Zhu K. (2015). A Time Space Translation Hypothesis for Vertebrate Axial Patterning. Semin. Cell Dev. Biol..

[7] Wacker S.A., Jansen H.J., McNulty C.L., Houtzager E., Durston A.J. (2004). Timed Interactions between the Hox Expressing Non-Organiser Mesoderm and the Spemann Organiser Generate Positional Information During Vertebrate Gastrulation. Dev. Biol..

[8] Wacker S., McNulty C., Durston A. (2004). The Initiation of Hox Gene Expression in Xenopus Laevis Is Controlled by Brachyury and Bmp-4. Dev. Biol..

[9] Smith W.C., Harland R.M. (1992). Expression Cloning of Noggin, a New Dorsalizing Factor Localized to the Spemann Organizer in Xenopus Embryos. Cell.

[10] Sasai Y., Lu B., Steinbeisser H., Geissert D., Gont L.K., De Robertis E. (1994). Xenopus Chordin: A Novel Dorsalizing Factor. Activated by Organizer-Specific Homeobox Genes. Cell.

[11] Smith W.C., Knecht A.K., Wu M., Harland R.M. (1993). Secreted Noggin Protein Mimics the Spemann Organizer in Dorsalizing Xenopus Mesoderm. Nature.

[12] Khokha M.K., Yeh J., Grammer T.C., Harland R.M. (2005). Depletion of Three Bmp Antagonists from Spemann’s Organizer Leads to a Catastrophic Loss of Dorsal Structures. Dev. Cell.

[13] Fang H., Marikawa Y., Elinson R.P. (2000). Ectopic Expression of Xenopus Noggin Rna Induces Complete Secondary Body Axes in Embryos of the Direct Developing Frog Eleutherodactylus Coqui. Dev. Genes Evol..

[14] Spemann H., Mangold H. (1924). Über Induktion Von Embryonalagen Durch Implantation Artfremder Organisatoren. Roux’s Arch. F. Entw. Mech..

[15] Dias A.S., de Almeida I., Belmonte J.M., Glazier J.A., Stern C.D. (2014). Somites without a Clock. Science.

[16] Kobayashi M., Toyama R., Takeda H., Dawid I.B., Kawakami K. (1998). Overexpression of the Forebrain-Specific Homeobox Gene Six3 Induces Rostral Forebrain Enlargement in Zebrafish. Development.

[17] Li Y., Allende M.L., Finkelstein R., Weinberg E.S. (1994). Expression of Two Zebrafish Orthodenticle-Related Genes in the Embryonic Brain. Mech. Dev..

[18] Mori H., Miyazaki Y., Morita T., Nitta H., Mishina M. (1994). Different Spatio-Temporal Expressions of Three Otx Homeoprotein Transcripts During Zebrafish Embryogenesis. Brain Res. Mol. Brain Res..

[19] Rhinn M., Lun K., Amores A., Yan Y.L., Postlethwait J.H., Brand M. (2003). Cloning, Expression and Relationship of Zebrafish Gbx1 and Gbx2 Genes to Fgf Signaling. Mech. Dev..

[20] Alexandre D., Clarke J.D., Oxtoby E., Yan Y.L., Jowett T., Holder N. (1996). Ectopic Expression of Hoxa-1 in the Zebrafish Alters the Fate of the Mandibular Arch Neural Crest and Phenocopies a Retinoic Acid-Induced Phenotype. Development.

[21] Hashiguchi M., Mullins M.C. (2013). Anteroposterior and Dorsoventral Patterning Are Coordinated by an Identical Patterning Clock. Development.

[22] Tucker J.A., Mintzer K.A., Mullins M.C. (2008). The Bmp Signaling Gradient Patterns Dorsoventral Tissues in a Temporally Progressive Manner Along the Anteroposterior Axis. Dev. Cell.

[23] Jacox L., Sindelka R., Chen J., Rothman A., Dickinson A., Sive H. (2014). The Extreme Anterior Domain Is an Essential Craniofacial Organizer Acting through Kinin-Kallikrein Signaling. Cell Rep..

[24] Dickinson A., Sive H. (2007). Positioning the Extreme Anterior in Xenopus: Cement Gland, Primary Mouth and Anterior Pituitary. Semin. Cell Dev. Biol..

[25] Sive H., Hattori K., Weintraub H. (1989). Progressive Determination During Formation of the Anteroposterior Axis in Xenopus Laevis. Cell.

[26] Scharf S.R., Gerhart J.C. (1983). Axis Determination in Eggs of Xenopus Laevis: A Critical Period before First Cleavage, Identified by the Common Effects of Cold, Pressure and Ultraviolet Irradiation. Dev. Biol..

[27] Kao K.R., Elinson R.P. (1988). The Entire Mesodermal Mantle Behaves as Spemann’s Organizer in Dorsoanterior Enhanced Xenopus Laevis Embryos. Dev. Biol..

[28] Faber J., Nieuwkoop P.D. (1994). Normal Table of Xenopus Laevis (Daudin): A Systematical and Chronological Survey of the Development from the Fertilized Egg Till the End of Metamorphosis.

[29] Cooke J., Smith J. (1989). Gastrulation and Larval Pattern in Xenopus after Blastocoelic Injection of a Xenopus-Derived Inducing Factor: Experiments Testing Models for the Normal Organization of Mesoderm. Dev. Biol..

[30] Nishimatsu S.I., Suzuki A., Shoda A., Murakami K., Ueno N. (1992). Genes for Bone Morphogenetic Proteins Are Differentially Transcribed in Early Amphibian Embryos. Biochem. Biophys. Res. Commun..

[31] Marom K., Levy V., Pillemer G., Fainsod A. (2005). Temporal Analysis of the Early Bmp Functions Identifies Distinct Anti-Organizer and Mesoderm Patterning Phases. Dev. Biol..

[32] Gammill L., Sive H. (2000). Coincidence of Otx2 and Bmp4 Signaling Correlates with Xenopus Cement Gland Formation. Mech. Dev..

[33] Kenyon K.L., Moody S.A., Jamrich M. (1999). A Novel Fork Head Gene Mediates Early Steps During Xenopus Lens Formation. Development.

[34] Blitz I.L., Cho K.W. (1995). Anterior Neurectoderm Is Progressively Induced During Gastrulation—The Role of the Xenopus Homeobox Gene Orthodenticle. Development.

[35] Von Bubnoff A., Schmidt J.E., Kimelman D. (1996). The Xenopus Laevis Homeobox Gene Xgbx-2 Is an Early Marker of Anteroposterior Patterning in the Ectoderm. Mech. Dev..

[36] Sive H.L., Cheng P.F. (1991). Retinoic Acid Perturbs the Expression of Xhox.Lab Genes and Alters Mesodermal Determination in Xenopus Laevis. Genes Dev..

[37] Zimmerman L.B., De Jesús-Escobar J.M., Harland R.M. (1996). The Spemann Organizer Signal Noggin Binds and Inactivates Bone Morphogenetic Protein 4. Cell.

[38] Clement J.H., Fettes P., Knöchel S., Lef J., Knöchel W. (1995). Bone Morphogenetic Protein-2 in the Early Development of Xenopus-Laevis. Mech. Dev..

[39] Dale L., Howes G., Price B.M., Smith J.C. (1992). Bone Morphogenetic Protein 4: A Ventralizing Factor in Early Xenopus Development. Development.

[40] Jones C.M., Lyons K.M., Lapan P.M., Wright C.V., Hogan B.L. (1992). Dvr-4 (Bone Morphogenetic Protein-4) as a Posterior-Ventralizing Factor in Xenopus Mesoderm Induction. Development.

[41] Schmidt J.E., Suzuki A., Ueno N., Kimelman D. (1995). Localized Bmp-4 Mediates Dorsal/Ventral Patterning in the Early Xenopus Embryo. Dev. Biol..

[42] Zhu K., Spaink H.P., Durston A.J. (2017). Collinear Hox-Hox Interactions Are Involved in Patterning the Vertebrate Anteroposterior (a-P) Axis. PLoS ONE.

[43] Goto K., Kamiya Y., Imamura T., Miyazono K., Miyazawa K. (2007). Selective Inhibitory Effects of Smad6 on Bone Morphogenetic Protein Type I Receptors. J. Biol. Chem..

[44] Hata A., Lagna G., Massagué J., Hemmati-Brivanlou A. (1998). Smad6 Inhibits Bmp/Smad1 Signaling by Specifically Competing with the Smad4 Tumor Suppressor. Genes Dev..

[45] Imamura T., Takase M., Nishihara A., Oeda E., Hanai J., Kawabata M., Miyazono K. (1997). Smad6 Inhibits Signalling by the Tgf-Beta Superfamily. Nature.

[46] Duboule D., Dollé P. (1989). The Structural and Functional-Organization of the Murine Hox Gene Family Resembles That of Drosophila Homeotic Genes. EMBO J..

[47] Graham A., Papalopulu N., Krumlauf R. (1989). The Murine and Drosophila Homeobox Gene Complexes Have Common Features of Organization and Expression. Cell.

[48] Lewis E.B. (1978). A Gene Complex Controlling Segmentation in Drosophila. Nature.

[49] Oliver G., Mailhos A., Wehr R., Copeland N.G., Jenkins N.A., Gruss P. (1995). Six3, a Murine Homologue of the Sine Oculis Gene, Demarcates the Most Anterior Border of the Developing Neural Plate and Is Expressed During Eye Development. Development.

[50] Kessel M., Gruss P. (1991). Homeotic Transformations of Murine Vertebrae and Concomitant Alteration of Hox Codes Induced by Retinoic Acid. Cell.

[51] Wellik D.M., Capecchi M.R. (2003). Hox10 and Hox11 Genes Are Required to Globally Pattern the Mammalian Skeleton. Science.

[52] Tour E., Pillemer G., Gruenbaum Y., Fainsod A. (2002). Gbx2 Interacts with Otx2 and Patterns the Anterior-Posterior Axis During Gastrulation in Xenopus. Mech. Dev..

