# Supplementary figures and images for: Patterning of the Vertebrate Head in Time and Space by BMP Signaling
Source: J Dev Biol. 2023 Jul 3;11(3):31. doi: 10.3390/jdb11030031 (PMC10366882; doi:10.3390/jdb11030031)

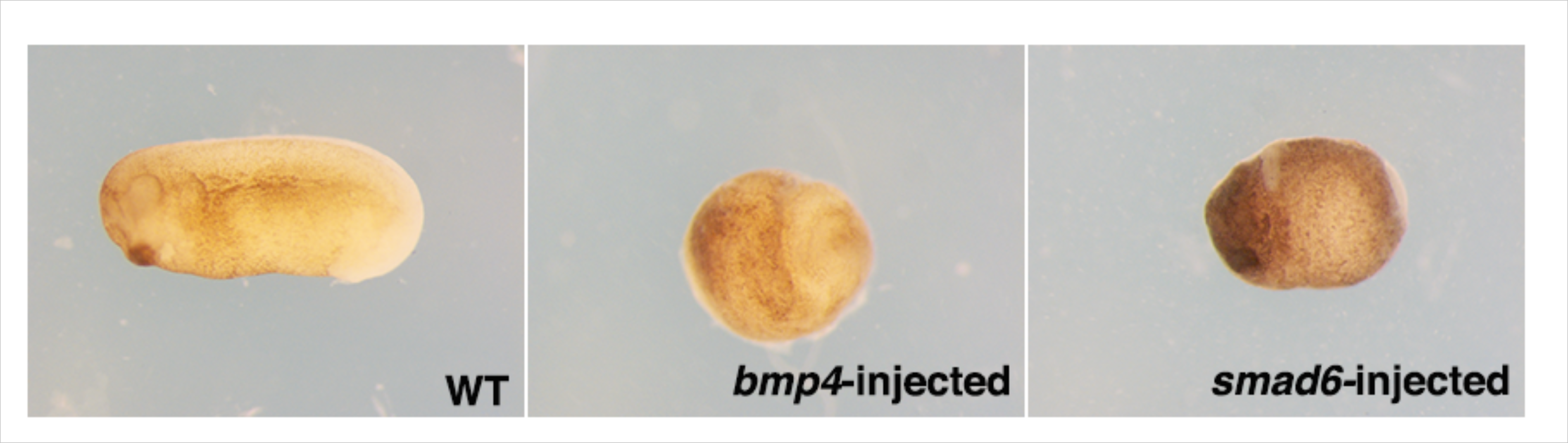

Supplement: Supplementary file 1 [file jdb-11-00031-s001.zip › Figure S1.tiff]

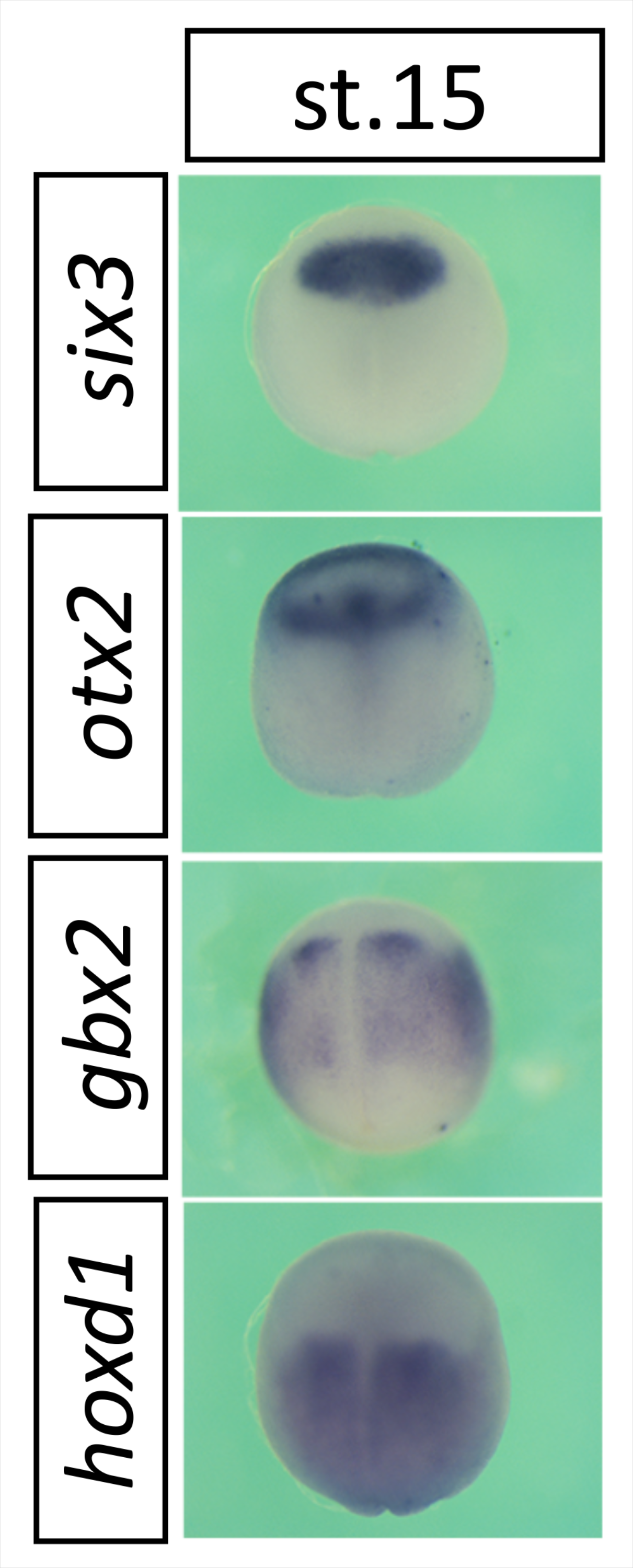

Supplement: Supplementary file 1 [file jdb-11-00031-s001.zip › Figure S2.tiff]
